# SERR Spectroelectrochemistry as a Guide for Rational Design of DyP-Based Bioelectronics Devices

**DOI:** 10.3390/ijms22157998

**Published:** 2021-07-27

**Authors:** Lidia Zuccarello, Catarina Barbosa, Edilson Galdino, Nikola Lončar, Célia M. Silveira, Marco W. Fraaije, Smilja Todorovic

**Affiliations:** 1 Instituto de Tecnologia Química e Biológica António Xavier, Universidade NOVA de Lisboa, Av. da República, 2780-157 Oeiras, Portugal; lidiazuccarello@itqb.unl.pt (L.Z.); catarina.barbosa@itqb.unl.pt (C.B.); edilson.galdino@itqb.unl.pt (E.G.); celiasilveira@itqb.unl.pt (C.M.S.); 2 Gecco Biotech, Nijenborgh 4, 9747AG Groningen, The Netherlands; n.loncar@gecco-biotech.com; 3 Molecular Enzymology, Groningen Biomolecular Sciences and Biotechnology Institute, University of Groningen, Nijenborgh 4, 9747AG Groningen, The Netherlands; m.w.fraaije@rug.nl

**Keywords:** DyP, SERR spectroelectrochemistry, immobilised enzymes, 3^rd^ generation biosensors

## Abstract

Immobilised dye-decolorizing peroxidases (DyPs) are promising biocatalysts for the development of biotechnological devices such as biosensors for the detection of H_2_O_2_. To this end, these enzymes have to preserve native, solution properties upon immobilisation on the electrode surface. In this work, DyPs from *Cellulomonas bogoriensis* (CboDyP), *Streptomyces coelicolor* (ScoDyP) and *Thermobifida fusca* (TfuDyP) are immobilised on biocompatible silver electrodes functionalized with alkanethiols. Their structural, redox and catalytic properties upon immobilisation are evaluated by surface-enhanced resonance Raman (SERR) spectroelectrochemistry and cyclic voltammetry. Among the studied electrode/DyP constructs, only CboDyP shows preserved native structure upon attachment to the electrode. However, a comparison of the redox potentials of the enzyme in solution and immobilised states reveals a large discrepancy, and the enzyme shows no electrocatalytic activity in the presence of H_2_O_2_. While some immobilised DyPs outperform existing peroxidase-based biosensors, others fail to fulfil the essential requirements that guarantee their applicability in the immobilised state. The capacity of SERR spectroelectrochemistry for fast screening of the performance of immobilised heme enzymes places it in the front-line of experimental approaches that can advance the search for promising DyP candidates.

## 1. Introduction

Dye-decolorizing peroxidases (DyPs) belong to a handful of oxidoreductase enzymes that can be explored for biotechnological applications. They efficiently oxidise a number of substrates (commonly referred to as oxidising substrates), such as synthetic azo- and anthraquinone-based dyes, metals, aromatic sulphides and phenolic and non-phenolic lignin units, with concomitant reduction of H_2_O_2_ at the heme *b* active site [1,2]. Therefore, they are potentially interesting for the development of H_2_O_2_ biosensors and devices for sensing and/or degradation of oxidising substrates of interest. The former are relevant for the detection of H_2_O_2_ in biomedical, environmental and food industry fields [3,4], while the latter can be applied for sensing the level and degradation of persistent dye pollutants in waste waters [2]. To that end, currently the most attractive platforms for the development of biosensors and bioelectrocatalytic devices are 3^rd^-generation electrochemical constructs. They rely on enzymes immobilised on biocompatible electrodes that undergo direct electron transfer (DET) with the electrode (i.e., electrochemical transducer), which acts as a source or a sink of electrons that drives the catalytic reaction in the presence of the substrate [5,6]. Crucial parameters that govern a rational design of 3^rd^-generation bioelectronic devices are the preservation of the solution structure, redox potential (E^0^) and catalytic parameters of the biocatalyst upon immobilisation, efficient DET between the enzyme and the electrode, good substrate accessibility to the active site of the enzyme and a long-term stability of the enzyme/electrode construct [7]. The only experimental approach that allows for the simultaneous and thorough structural characterization and redox potential determination of the immobilised enzyme in situ relies on surface-enhanced resonance Raman (SERR) spectroelectrochemistry. The SERR spectra of heme proteins immobilised on plasmonic Ag electrodes, obtained by excitation in resonance with the Soret band of the porphyrin, display core-size marker bands (ν_4_, ν_3_, ν_2_ and ν_10_) of the immobilised molecules, the frequencies of which are indicative of the redox, spin and coordination state of the heme iron [8,9,10,11]. A comparison of the resonance Raman (RR) spectra of the enzyme, which disclose equivalent spin/redox properties in solution, with the SERR spectra of the immobilised enzymes, can sensitively reveal eventual immobilisation-induced structural changes on the level of the active site [8,10,12,13,14]. These structural changes that frequently occur upon attachment of the enzyme to electrodes are actually the most common cause for the failure of 3^rd^-generation devices [14]. The catalytic performance of immobilised enzymes can be simultaneously monitored by electrochemical methods [7]. This experimental approach that couples SERR with electrochemistry has been used to evaluate the potential of several interesting enzymes for the development of biotechnological applications; these include cytochrome P450, nitrite reductases, microperoxidases and, more recently, DyP peroxidases [13,14,15,16,17,18,19,20]. In particular, DyP from *Pseudomonas putida* (PpDyP) has been shown to be an excellent candidate for the construction of 3^rd^-generation H_2_O_2_ biosensors [21]. PpDyP-based constructs are actually capable of outperforming all commercially available H_2_O_2_ biosensors, including the recently developed horseradish peroxidase (HRP)-based devices that employ advanced nanostructures, such as carbon nanotubes, nano-dots and ceramic and gold nanostructures [22,23,24].

Here, we define a pipeline of requirements that allow for the stepwise evaluation of the aptness of several DyPs for the rational development of biosensors and/or devices for bioelectrocatalytic degradation of chemically inert dyes. We use SERR spectroelectrochemistry to probe the structural integrity and redox potential of the enzymes upon immobilisation on biocompatible electrodes and amperometric/voltammetric methods to monitor the electrocatalytic activity of the constructs that carry enzymes immobilised in the native state. We discuss the outcomes obtained for DyPs from different bacteria in light of our previously published work on PpDyP [15,21].

## 2. Results

### 2.1. Evaluation of Structural Integrity of DyPs upon Immobilisation

**Figure 1:**
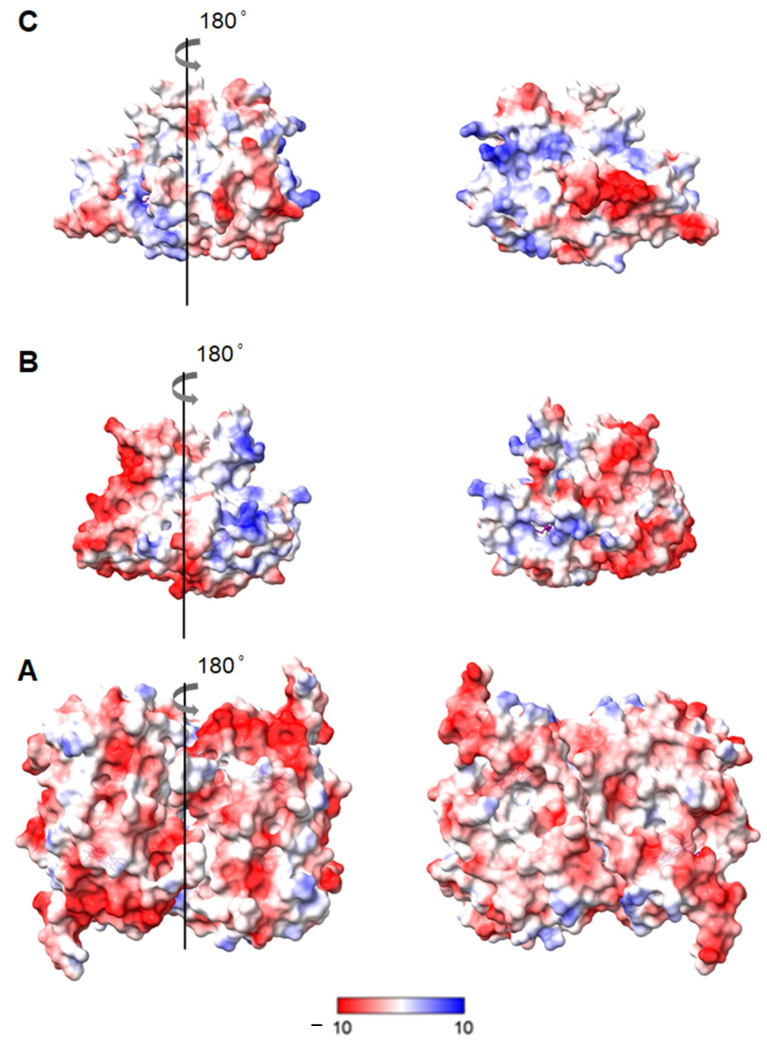
Electrostatic surface potential of (**A**) *Cellulomonas bogoriensis* (CboDyP) (dimer; PDB code: 6QZO), (**B**) *Thermobifida fusca* (TfuDyP) (monomer; PDB code: 5FW4) and (**C**) *Streptomyces coelicor* (ScoDyP) (monomer; PDB code: 4GT2). Surface potential: −10 to +10 kT/e; the red colour indicates negatively charged regions; the blue colour indicates positively charged regions. Figures were prepared with ChimeraX [25].

Surface charge distribution analysis. The immobilisation of DyPs from *Cellulomonas bogoriensis* (CboDyP), *Streptomyces coelicor* (ScoDyP) and *Thermobifida fusca* (TfuDyP) was attempted on Ag electrodes coated with alkanethiol self-assembled monolayers (SAMs) to ensure biocompatibility. The choice of a SAM for each studied enzyme followed an initial consideration of the surface charge distribution of the DyPs (Figure 1). CboDyP has a predominantly negative surface charge, with several relatively well-defined patches, intertwined with hydrophobic surface residues. This suggests that a positively charged surface (e.g., NH_2_-terminated SAMs) can favour electrostatic interactions with the enzyme. The TfuDyP surface appears quite polarized, with a region of predominantly negative surface charges counter-balanced with hydrophobic and positively charged amino acid residues. In ScoDyP, opposite-charge regions are, together with hydrophobic residues, more homogeneously distributed over the surface. Accordingly, positively (NH_2_) as well as hydrophobic (CH_3_) and negatively (COO^−^) charged SAMs (either pure or mixed) were tested to optimise the immobilisation conditions (*vide infra*).

**Figure 2:**
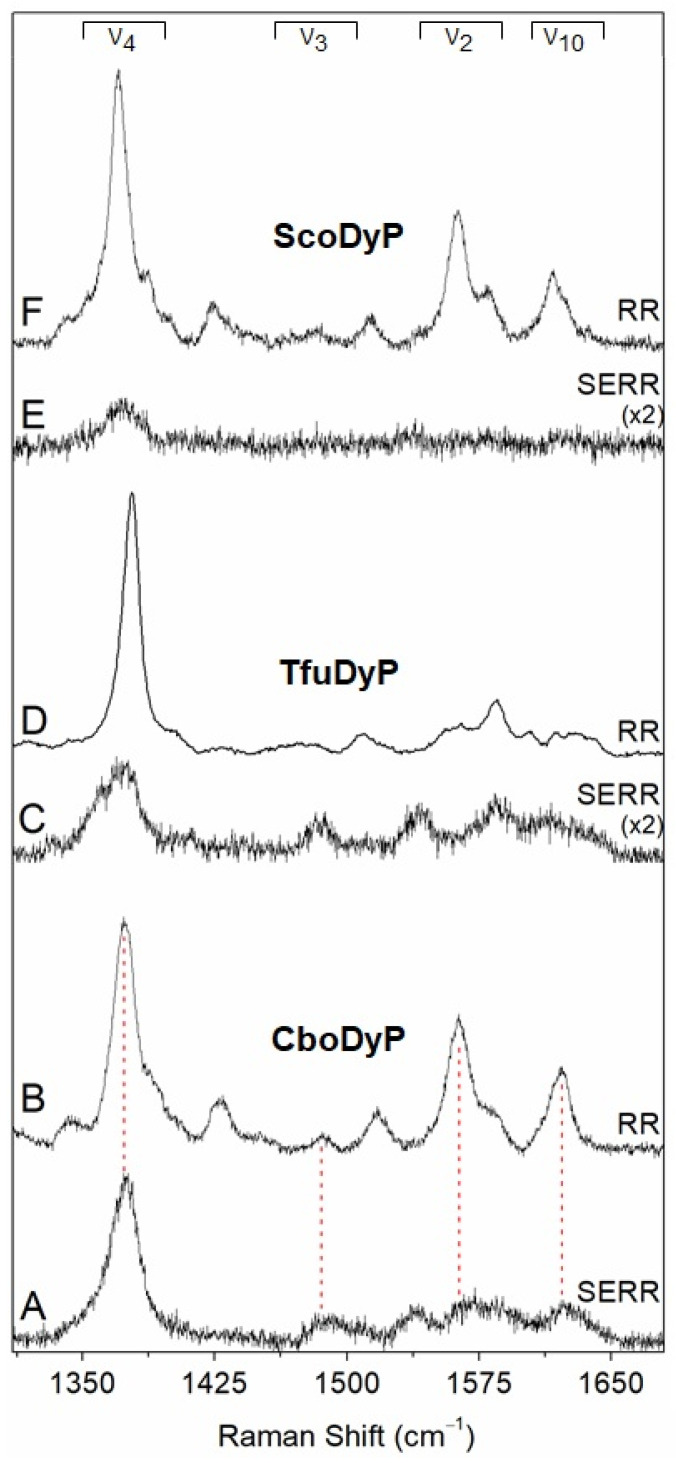
Resonance Raman (RR) and surface-enhanced resonance Raman (SERR) spectra of ferric dye-decolorizing peroxidases (DyPs): (**A**) SERR spectrum of CboDyP immobilised on Ag electrodes coated with 1-undecanethiol:11-amino-1-undecanethiol hydrochloride (M:M, 2:1) self-assembled monolayer (SAM) at pH 7, recorded at +250 mV electrode poised potential; (**B**) RR spectrum of CboDyP in solution at pH 8; (**C**) SERR spectrum of TfuDyP immobilised on Ag electrodes coated with 1-undecanethiol: 11-amino-1-undecanethiol hydrochloride SAM (M:M, 2:1) at pH 7, recorded at +350 mV electrode poised potential; (**D**) RR spectrum of TfuDyP in solution at pH 7.5; (**E**) SERR spectrum of ScoDyP immobilised on a 1-undecanethiol SAM at pH 7, recorded at +250 mV electrode poised potential; (**F**) RR spectrum of ScoDyP in solution at pH 8. The RR spectra of CboDyP (at 21 °C) and ScoDyP (at −50 °C) were acquired with 405 nm excitation, and the RR spectrum of TfuDyP (at 21 °C) was obtained with 413 nm excitation. All SERR spectra were acquired with 405 nm excitation at 21 °C (*vide infra* Materials and Methods).

DyPs in solution. In order to provide a reference for the studies of immobilised enzymes, the RR spectra of as purified, ferric DyPs were measured in solution. The high frequency region of the RR spectra of CboDyP show oxidation (ν_4_) and oxidation/spin state (ν_3_ and ν_2_) marker bands at 1371, 1481 and 1561 cm^−1^ at pH 8, which are indicative of a uniform and homogeneous 6-coordinated high-spin (6cHS) population (Figure 2B) [26].

The RR features of TfuDyP at pH 7.5 reveal ν_4_, ν_3_ and ν_2_ marker bands at 1378, 1509 and 1585 cm^−1^, characteristic of a single ferric 6-coordinated low-spin (6cLS) population [26] (Figure 2D). The RR spectrum of ScoDyP is indicative of a presence of a 6cHS population at pH 8, with ν_4_, ν_3_ and ν_2_ bands at 1370, 1481 and 1562 cm^−1^, respectively (Figure 2F).

Immobilised DyPs. In the next step, the immobilisation conditions were tested for the studied DyPs. The SAM-coated working Ag electrodes were incubated in the enzyme solution during variable time intervals (*vide infra*), and the SERR spectrum of each DyP/SAM Ag construct was measured in a three-electrode spectroelectrochemical cell, equipped with reference and counter electrodes. The immobilisation of CboDyP, TfuDyP and ScoDyP was attempted using pure CH_3_, NH_2_ and COO^−^-terminated SAMs, as well as CH_3_ mixed with NH_2_ or COO^−^-terminated alkanethiols. For CboDyP and ScoDyP, pure OH and mixed OH/CH_3_-terminated SAMs were also employed.

CboDyP and TfuDyP can be successfully immobilised on mixed hydrophobic and positively charged surfaces, with CH_3_ and NH_2_-terminated SAMs in a 2:1 concentration ratio, as indicated by the presence of marker bands in the high frequency region of the respective SERR spectra (Figure 2A,C). Under all other tested conditions either residual or no SERR signals are observed (Figure 2E and Figure S1), which could be due to unfavourable interactions and/or low enzyme loading. Mixed CH_3_/NH_2_-terminated SAMs were, therefore, selected for further studies of TfuDyP and CboDyP, while no suitable platform for attachment of ScoDyP could be identified. The optimal SAM composition for the immobilisation of TfuDyP and CboDyP suggests that an interplay of hydrophobic and electrostatic interactions is required, which is in agreement with the presence of negatively charged patches and hydrophobic residues at the surface of these enzymes (cf. Figure 1).

**Figure 3:**
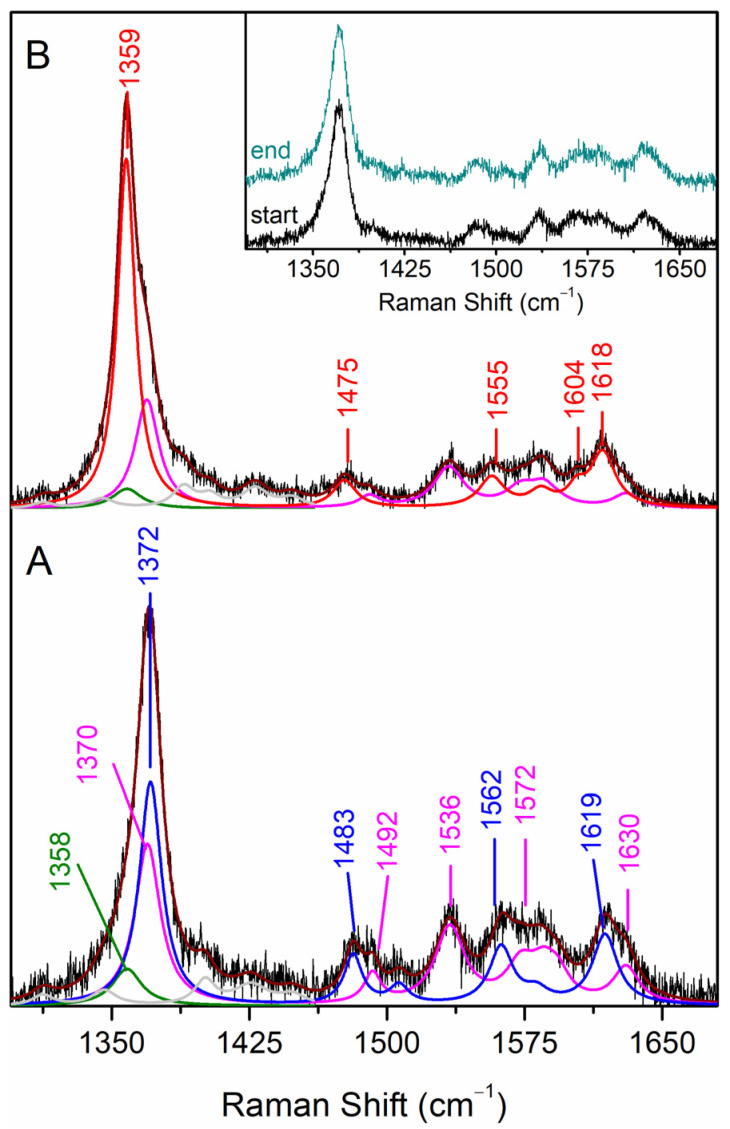
SERR spectra of CboDyP immobilised on Ag electrodes coated with 1-undecanethiol: 11-amino-1-undecanethiol hydrochloride SAM (M:M, 2:1) recorded at potentials poised at (**A**) +250 mV and (**B**) −200 mV. The component spectra represent the overall fit (dark red), ferric 6cHS (blue), redox-inactive ferric 5cHS (magenta), ferrous 6cHS (red) and redox-inactive ferrous (green) species and non-assigned bands (grey). Inset: SERR spectra acquired at the electrode potential poised of +250 mV before and after the electrode was poised at four different potentials. All spectra were obtained with 405 nm excitation at pH 7 and 21 °C.

The SERR spectra of CboDyP attached to the electrode at a positive poised potential of +250 mV vs. normal hydrogen electrode (NHE; all potential values in this work are quoted vs. NHE) show subtle differences in comparison with the RR spectra of ferric CboDyP. The component analysis of the SERR spectra, in which the individual heme species are fitted to the experimental spectra, reveals that multiple heme populations co-exist at the electrode surface (Figure 3A), while a uniform 6cHS species is observed in solution. The major population is assigned to the native 6cHS heme species, with spectral parameters comparable to those found in the RR spectra (ν_4_, ν_3_ and ν_2_ bands at 1372, 1483 and 1562 cm^−1^, respectively). The widths of the SERR component bands are ca. 1–2 cm^−1^ larger than those observed in the RR spectra, which could suggest the orientational distribution of the enzyme molecules on the electrode. The ν_4_, ν_3_, and ν_2_ bands of the second population, found at 1370, 1492 and 1572 cm^−1^, respectively (representing ca. 35% of the area of the ν_4_ band) are characteristic of 5cHS heme species, in which the 6th axial heme Fe position is vacant. The third minor species comprising ca. 10% of ν_4_ mode is centred at 1358 cm^−1^, which is indicative of reduced protein; owing to a low amount of the ferrous population, ν_3_ and ν_2_ bands could not be identified in the spectra.

The SERR spectra of TfuDyP, obtained at electrode potential poised at +350 mV, are clearly different from the RR spectra of the ferric enzyme in solution (Figure 2C,D). The ν_3_ region has a major component centred at 1484 cm^−1^, consistent with a presence of a HS heme species, which in solution has only been identified at acidic pH values. At pH 3.5, TfuDyP in solution undergoes a complete transition to HS species. This becomes evident from the presence of ν_3_ modes at 1481 cm^−1^ (6cHS) and 1493 cm^−1^ (5cHS) and downshifted ν_4_ (1373 cm^−1^) [26]. This conformational change from 6cLS to HS species coincides with the transition from a catalytically impaired state at pH 7 to a catalytically active enzyme at pH 3.5 in solution [26,27]. However, the frequencies, bandwidths and relative intensities of the heme marker bands observed in the SERR spectra are distinct from the HS species previously identified in solution. Furthermore, a significant amount of the enzyme appears to be in a ferrous state, as indicated by the presence of a ν_4_ mode centred at 1355 cm^−1^, even at a positive poised electrode potential (+350 mV). These findings indicate that TfuDyP undergoes alterations at the level of the heme environment upon attachment to the employed SAM/Ag surfaces.

Taken together, these results show that among the studied DyPs, CboDyP appears to be the best candidate for further studies, as a large amount of the enzyme retains its native properties upon immobilisation.

### 2.2. Probing Redox Activity, Reversibility and Electronic Coupling of the Immobilised CboDyP

In order to evaluate the efficiency of the DET of the immobilised enzyme, the electrode potential was switched from a positive to a negative value, at which CboDyP was expected to be fully reduced. At the most negative potential applicable to the SAM-coated Ag electrode, i.e., −400 mV, which is defined by the reductive desorption of the alkanethiol monolayer, the immobilised CboDyP appears to be reduced. The redox transition is fast and reversible for several redox steps, as concluded from identical SERR spectra measured at e.g., +250 mV before and after the application of up to four different poised potentials to the electrode (Figure 3, inset). All further measurements were performed by taking this finding into consideration (i.e., up to four SERR spectra were recorded from a single electrode at different potentials). However, the immobilised CboDyP is not fully reduced at −200 mV electrode poised potential, which is evident from an asymmetric redox sensitive ν_4_ band, indicating the presence of oxidised species (Figure 3B). In fact, no further reduction is observed at potentials poised below −150 mV. The component analysis of the SERR spectra reveals the presence of two populations with ν_4_ bands at 1359 and 1370 cm^−1^ characteristic of ferrous and ferric species, respectively (Figure 3B). The marker bands of the major 6cHS species (ν_4_ and ν_3_ at 1359 and 1475 cm^−1^, respectively) are ca. 10 cm^−1^ upshifted in comparison to the SERR spectra at +250 mV electrode poised potential, as expected for the ferrous heme. The spectral parameters of the minor ferric population are identical to those observed for the 5cHS species at +250 mV, leading us to conclude that it represents a non-native, redox-inactive species that is formed upon the attachment of CboDyP to the electrode.

### 2.3. Assessement of the Redox Potential and Catalytic Efficiency of the Immobilised CboDyP

**Figure 4:**
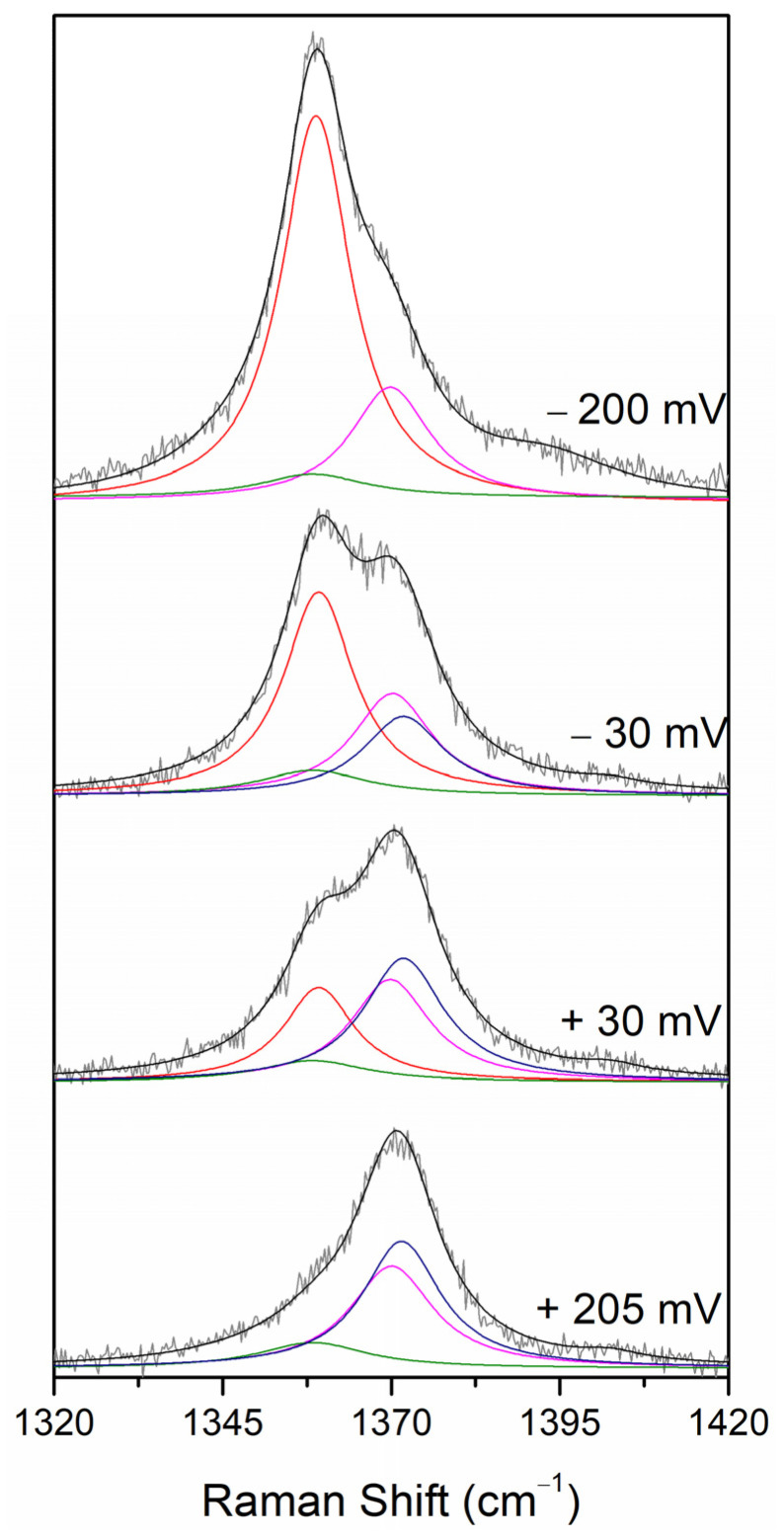
The ν_4_ band region of the SERR spectra of CboDyP immobilised on Ag electrodes coated with 1-undecanethiol and 11-amino-1-undecanethiol hydrochloride SAM (M:M, 2:1), recorded at several selected electrode potentials. The component spectra represent the overall fit (black) and ferric 6cHS (blue), ferric redox-inactive 5cHS (magenta), ferrous 6cHS (red) and ferrous redox-inactive (green) species. Spectra were obtained with 405 nm excitation at pH 7 and 21 °C (*vide infra* Materials and Methods).

In the next step, SERR spectroelectrochemical titrations were employed to determine the E^0^ of the Fe^3+^/Fe^2+^ redox couple of the immobilised CboDyP. The spectra were measured at a series of poised potentials, and the contributions of the native 6cHS ferric and ferrous species were determined from the component analysis of the potential-dependent SERR spectra (Figure 4). Note that the component analysis took into account the presence of the redox-inactive ferric (5cHS, ν_4_ 1370 cm^−1^) and ferrous (ν_4_ 1358 cm^−1^) species (designated by magenta and green traces in Figure 4), which remain constant upon variation of the electrode potential. Due to the presence of multiple species (i.e., high complexity) and the poor S/N ratio of the spectra, only the ν_4_ mode was fitted. A similar approach was earlier successfully adopted for the analysis of the redox behaviour of multiple heme and/or multiple spin species containing proteins [15,17].

**Figure 5:**
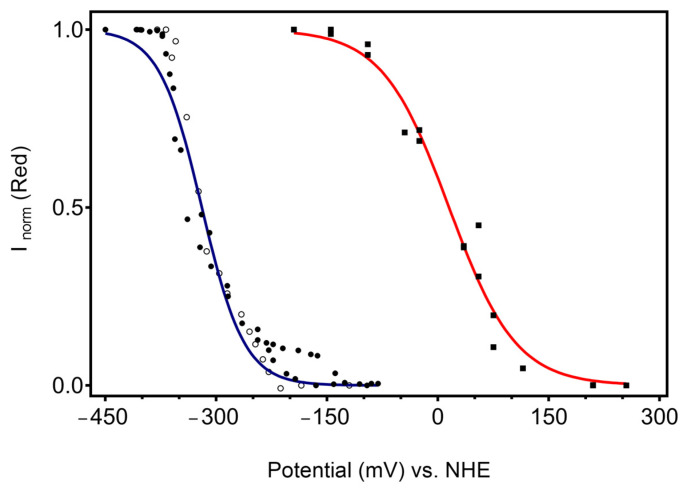
Redox titrations of CboDyP in solution and immobilised states. Left curve: potentiometric titration in solution monitored by UV–VIS spectroscopy. The circles represent the normalized absorption of the reduced population at 430 nm as a function of the solution potential for three independent experiments. Solid (and empty) symbols represent titrations by stepwise reduction (and oxidation) of the ferric (and ferrous) enzyme. Right curve: SERR spectroelectrochemical titration of CboDyP immobilised on electrodes coated with 1-undecanethiol and 11-amino-1-undecanethiol hydrochloride SAM (M:M, 2:1). The squares represent the relative contribution of the ferrous 6cHS population estimated from the ν_4_ band (1359 cm^−1^) at variable electrode potentials for seven independent experiments. Solid lines represent fits of the Nernst equation to the experimental data points, yielding for solution enzyme, E^0^_sol_ = −320 mV, *n* = 0.9, R^2^ = 0.9698 (blue), and for immobilised enzyme, E^0^_imm_ = +15 mV, *n* = 0.6 and R^2^ = 0.9797 (red).

The potential dependence of relative spectral contributions of the native 6cHS ferrous species follows a sigmoid shape. The fit of the Nernst equation to the data reveals the redox potential of the immobilised CboDyP (E^0^_imm_ = 15 ± 14 mV) and the number of exchanged electrons (*n* = 0.6) (Figure 5, right trace). The redox potential is, nevertheless, distinctively different from that in solution (E^0^_sol_ = −320 ± 12 mV; *n* = 0.9), estimated from potentiometric titrations using sodium dithionite as reducing agent (Figure 5, left trace). The redox reaction is fully reversible in solution, as demonstrated by the titration undertaken in the opposite direction, in which the fully sodium dithionite-reduced CboDyP was oxidised in a stepwise manner by potassium ferricyanide, which is revealed by the same E^0^_sol_ (Fe^3+^/Fe^2+^).

The electrocatalytic reduction of H_2_O_2_ by the immobilised CboDyP was evaluated by cyclic voltammetry (CV) and amperometry at pH_opt_ 5 [28] and pH 7, directly in the SERR cell. A broad range of substrate concentrations was tested (8 μM to 1.7 mM final concentration of H_2_O_2_) in a number of independent measurements. No catalytic signal is detected in CV experiments in any of the employed conditions, since the measured currents do not differ significantly from the control, i.e., in the presence/absence of H_2_O_2_ with the working electrode carrying attached CboDyP (Figure S2A) or in the presence/absence of the immobilised enzyme on the working electrode and H_2_O_2_ in the solution. In the next step, more sensitive, amperometric assays were performed, in which the response of the immobilised enzyme to H_2_O_2_ was evaluated by stepwise injections of the deoxygenated stock solution of the substrate. The amperometric response of CboDyP immobilised on electrodes poised at −100 mV potential is comparable to that of the control assay (Figure S2B), in which the working electrode carries no enzyme. Therefore, we have concluded that the immobilised CboDyP displays no electrocatalytic activity under the employed experimental conditions. These results indicate that despite being immobilised in an apparently native-like state, CboDyP does not retain its catalytic and redox properties upon attachment to the modified electrodes.

## 3. Discussion

Heme-containing peroxidases, including the best studied HRP, as well as cytochrome *c* peroxidase (C*c*P), chloroperoxidase and lactoperoxidase, show a significant potential as biocatalysts for the development of 3^rd^-generation biotechnological devices [29,30]. Bacterial DyPs are also regarded as promising candidates, since they possess broad substrate specificity, they can easily be heterologously overexpressed in non-glycosylated form, and their properties improved by protein engineering [1,2,31,32,33,34]. However, the progress in the development of peroxidase-based applications is lagging behind the expectations, due to the difficulties in transferring the enzyme catalytic efficiency in solution to the immobilised state and the lack of experimental approaches for fast screening of enzyme/electrode constructs in situ [30]. The enzyme structural integrity, its redox properties and reversibility of the redox reaction, catalytic efficiency and stability need to be preserved upon immobilisation, and the efficient electronic communication with the electrode has to be ensured [7,30]. Therefore, sensitive methods that can probe these features to allow for a rational design of peroxidase-based devices with optimised performance, are highly required [21,29].

### 3.1. Structural Integrity upon Immobilisation

Due to a flexible distal heme cavity, peroxidases are capable of adopting distinct heme configurations in the resting ferric state. These include catalytically competent 5cHS and 6cHS and quantum mechanically mixed-spin (QS) states, which frequently co-exist in solution [35,36]. Unlike classical peroxidases, DyPs often possess a significant amount of 6cLS population [16,26], which, due to the occupied 6th axial iron position, cannot bind H_2_O_2_ at the heme active site and is therefore catalytically incompetent [16]. Probing of the heme configurations of an enzyme upon attachment to solid supports exclusively relies on SERR spectroscopy that can sensitively identify different spin populations and eventual immobilisation-induced alterations. Previously, SERR spectroscopy has been used to monitor the structural integrity of the heme active site(s) in immobilised cytochrome P450, nitrite reductases, heme oxidases, microperoxidases, as well as DyPs from *Pseudomonas putida* and *Bacillus subtilis* (PpDyP and BsDyP) among others, offering molecular details that help rationalise the functional failure of some of these enzymes in the immobilised state [13,14,15,16,17,18,19,20]. For instance, BsDyP that contains a mixture of HS and LS populations in solution reveals an increase of catalytically inactive 6cLS species upon immobilisation [16]. On the other hand, the structural integrity of the heme pocket is preserved upon the immobilisation of PpDyP, as the frequencies, bandwidths and relative ratios of bands characteristic of 5cHS and 5cQS species are equal in solution RR and in SERR spectra [15]. Here, we show that CboDyP, which is characterized by uniform 6cHS population in solution, adopts multiple conformations upon immobilisation. The most representative are the native 6cHS species (with a spectral contribution of approximately 55%), which does not undergo apparent structural alterations, and the non-native ferric redox-inactive 5cHS species (ca. 35%). TfuDyP could not be immobilised in the native state, while ScoDyP could not be attached to any of the tested SAMs.

### 3.2. Redox Properties upon Immobilisation and Electronic Communication

Heme-containing peroxidases typically have a negative redox potential of the Fe^3+^/Fe^2+^ couple, which guarantees that the ferric form, that is required for H_2_O_2_-mediated two-electron oxidation of ferric peroxidases to Compound I, is stable under physiological conditions [37]. Although not directly involved in the catalytic cycle, the E^0^ (Fe^3+^/Fe^2+^) is considered to be a good indicator of the redox properties of catalytically relevant redox couples (i.e., Fe^3+^/Compound I, Compound I/Compound II and Compound II/Fe^3+^), which govern the catalytic activity of peroxidases [37]. Moreover, the molecular factors that impact E^0^ (Fe^3+^/Fe^2+^) in heme proteins in general, i.e., the electron donor properties of the axial Fe ligands, the polarity of the protein environment and the electrostatic interactions between the heme Fe and polar and charged amino acid residues and the solvent [37], have been suggested to influence the catalytically relevant redox couples in the same manner.

Here, we demonstrate that the value of E^0^ (Fe^3+^/Fe^2+^) of CboDyP in solution (−320 mV) is among the lowest reported for DyPs. This is not an advantage for potential applications. Namely, as judged by considerations of Gibbs free energy only, a more positive redox potential implies a broader range of oxidisable substrates and consequently higher enzyme applicability in biotechnology. So far, the reported E^0^ (Fe^3+^/Fe^2+^) of DyPs span over a broad range: BsDyP (−40 mV) [16] and variants (−70 to −120 mV) [33], PpDyP (−260 mV) [15], DyP 2 from *Amycolatopsis* sp. 75iv2 (−85 mV) [38], *Thermomonospora curvata* DyP (TcDyP) (−136 mV) and variants (−130 to −210 mV) [39], *Klebsiella pneumoniae* DyP (KpDyP) (−350 mV) [40] and variants (−300 to −350 mV) [41]. It is tempting to relate different values of E^0^ (Fe^3+^/Fe^2+^) with distinct physiological functions of DyPs, with higher E^0^ reflecting a physiological requirement for more versatile enzymes. However, so far, there have been neither enough data nor sufficient evidence to support subfamily-related correlation between the two.

The immobilisation of enzymes can cause large shifts in their redox potentials. The reported E^0^ (Fe^3+^/Fe^2+^) values of immobilised HRP vary significantly, ranging between −450 and +110 mV, and are strongly affected by the choice of electrode material and immobilisation strategies [13]. Here, we show that in the case of CboDyP, a significant amount of the enzyme is immobilised in a non-native state. The portion of the enzyme that shows no apparent structural alterations, nevertheless, does not preserve its solution redox properties, with the E^0^_sol_ vs. E^0^_imm_ differing about 300 mV. Previously, the E^0^ (Fe^3+^/Fe^2+^) of the catalytically competent 6cHS population in BsDyP was determined by SERR redox titration, revealing a relatively insignificant modulation of the redox potential in comparison with the solution value (E^0^_sol_ vs. E^0^_imm_ differ approximately ±50 mV) [16]. In the case of PpDyP, E^0^_sol_ (−260 mV) is, within the error of determination, the same as the E^0^_imm_ (−300 mV), with the latter being comparable between the two redox active species, i.e., 5cHS and 5cQS [15]. We attribute the large discrepancy in E^0^_sol_ vs. E^0^_imm_ of 6cHS population in CboDyP to a non-uniform orientation of the enzyme molecules on the electrode surface, revealed by the enlarged width of SERR bands and also the broad redox transition with *n* < 1. This heterogeneous orientation may be related with the non-specificity of hydrophobic interactions between the enzyme and the modified electrode, as the CH_3_-terminated SAM constitutes the major portion of the immobilisation surface. Another factor that likely contributes to the large upshift of E^0^_imm_ is the lowering of polarity in the heme cavity upon immobilisation. As predicted by the Kassner relation, a decreased hydration of the heme environment lowers the local dielectric constant and can account for up to 200 mV upshift of redox potential in heme proteins [42]. One can envisage subtle immobilisation-induced conformational changes that propagate to the active site and further increase the hydrophobicity of CboDyP heme cavity, which is not necessarily accompanied by the exchange of heme ligands that can be detected spectroscopically; however, at this point, no evidence can be provided to support this hypothesis.

### 3.3. Probing Electrocatalytic Activity upon Immobilisation

A comparable electrocatalytic activity of an immobilised DyP with its catalytic activity in solution is the ultimate requirement that the enzyme needs to satisfy in order to be considered for applications. The electrocatalytic activity, substrate affinity (K_M_) and substrate inhibition (K_i_) can be readily obtained by CV and amperometry. In the case of immobilised PpDyP and variants, apparent Michaelis–Menten constant values, K_M_, are 2–5 times higher than in solution [15,21,31,32,34]. This is common for enzymes immobilised on electrode surfaces, due to frequently limited diffusion of the substrate to the enzyme’s active site [30]. The catalytic efficiency, measured by catalytic currents in the presence of substrate, is also typically reduced in the immobilised state. Surprisingly, substrate inhibition in some cases can actually be improved upon the attachment of the enzyme to a biocompatible electrode, as demonstrated for PpDyP and its variant carrying E188K and H125Y mutations. Substrate inhibition between 0.7 and 0.9 mM H_2_O_2_ characterize the solution state of both PpDyP and its variant, while no inhibition is detected upon their immobilisation [21]. The immobilised BsDyP shows only residual catalytic currents in the presence of H_2_O_2_ [16]. Similarly, for CboDyP, no catalytic activity could be measured in the immobilised state. Most likely, the altered redox properties, together with an orientation that is unfavourable for H_2_O_2_ binding, are responsible for the lack of catalytic activity of the immobilised enzyme. This is actually not uncommon; among a number of studied DyPs, e.g., DrDyP, BsDyP [16], TfuDyP and ScoDyP, only immobilised PpDyP and one of its variants fulfil all necessary criteria to be considered excellent candidates for the development of 3^rd^-generation biotechnological devices [15,21].

Taken together, our findings emphasize the importance of the fast and straightforward evaluation of the potential of an enzyme for the development of 3^rd^-generation bioelectronic devices. We demonstrate that the coupling of SERR spectroelectrochemistry and electrochemistry can indeed provide a complete picture about the structure and activity of immobilised enzymes; there is no matching alternative in other experimental approaches. As such, SERR spectroelectrochemistry has the capacity to ensure faster advances in the rational design of DyP-based devices by pinpointing the best candidates and/or parameters that need to be improved in the process of their construction, which is clearly a step forward in comparison with the commonly employed empirical strategies for the development of 3^rd^-generation bioelectronic devices.

## 4. Materials and Methods

### 4.1. Enzymes and Reagents

The overexpression of the recombinant enzymes, *Cellulomonas bogoriensis* DyP (CboDyP), *Thermobifida fusca* DyP (TfuDyP) and *Streptomyces coelicolor* (ScoDyP), was performed using *Escherichia coli* as the expression host and following previously optimised procedures. Enzyme purification was performed as previously described [27,28]. The purified enzymes were stored at −80 °C in 50 mM KPi and 150 mM NaCl buffer solution at pH 8.

Chemicals were purchased from Sigma-Aldrich (St. Louis, MO, USA) and were of the highest purity grade available. Solutions were prepared using deionized water from a Milli-Q^®^ Water System (Merck Millipore, Burlington, MA, USA).

### 4.2. Enzyme Immobilisation

Silver electrodes were electrochemically roughened as previously described [9] and subsequently immersed for 16–24 h in 1 mM ethanolic solutions of alkanethiols to form a SAM. For pure SAMs, the respective alkanethiol was dissolved in ethanol to yield a 1 mM solution. For mixed SAMs, 1 mM of one alkanethiol was mixed with 1–3 mM of another one. The following SAMs were tested: pure 1-undecanethiol, 11-amino-1-undecanethiol hydrochloride, 11-mercaptoundecanoic and 11-mercapto-1-undecanol; and mixed 1-undecanethiol/11-mercaptoundecanoic acid (M:M, 1:1), 1-undecanethiol/11-mercapto-1-undecanol (M:M, 3:1), 1-undecanethiol/11-amino-1-undecanethiol hydrochloride (M:M, 1:1; M:M, 2:1; and M:M, 3:1). Prior to SERR experiments, the DyPs were immobilised on the SAM-coated Ag electrodes, either by immersion into 12.5 mM KPi and 12.5 mM K_2_SO_4_, pH 7, containing DyP (final concentration: ca. 0.3–0.5 µM) for 15–30 min or by injecting the enzyme into the SERR cell containing the same buffer (10 mL) and with the electrode poised at +250 mV vs. NHE. All potentials in this work were referenced to the NHE.

### 4.3. RR Spectroscopy

RR and SERR spectra were acquired with a Raman spectrometer (Jobin Yvon U1000, Edison, NJ, USA), equipped with a 1200 lines/mm grating and a liquid-nitrogen-cooled CCD detector, which was coupled to a confocal microscope. An Olympus 20× objective was used for laser focusing onto the sample and light collection in the backscattering geometry. Spectra were measured using a 405 nm diode laser (Toptica Photonics AG, Munich, Germany).

The RR spectra of CboDyP and TfuDyP were measured as previously described [26]. ScoDyP spectra were acquired at low temperature using ca. 2 μL of the enzyme sample placed in a microscope stage (Linkham THMS 600, Tadworth, UK) cooled to the desired temperature with liquid N_2_.

RR experiments were performed with a 1.8 mW laser power and a 120 s accumulation time. SERR experiments were performed with a 1.3 mW laser power and 30–40 s accumulation time. Up to 16 spectra were co-added in each measurement to improve the signal-to-noise ratio (S/N). All spectra were subjected to polynomial baseline subtraction; the positions and widths of Raman bands were determined by component analysis as described previously [43].

### 4.4. Redox Potential Determination in the Immobilised State

Potential-controlled SERR experiments were performed using a home-built spectroelectrochemical cell equipped with a Ag/AgCl (3 M, KCl) reference electrode and a platinum wire counter electrode. The electrodes were poised to potentials between +350 and −400 mV vs. NHE. The electrode potentials were controlled using a Princeton Applied Research 263A potentiostat (Oak Ridge, TN, USA). The experiments were carried out in argon-purged supporting electrolyte, 12.5 mM KPi and 12.5 mM K_2_SO_4_, pH 7, to avoid the formation of O_2_ reduction products that might interact with the immobilised enzyme. The enzyme-loaded working electrodes were kept under constant rotation (1500 rpm) to prevent the prolonged exposure of individual enzyme molecules to laser irradiation. To prevent enzyme degradation during the titration assays, up to four spectra at different potentials were acquired per each electrode. Enzyme reduction was monitored by the evolution of the ferrous 6cHS population determined by the component analysis. The redox parameters were obtained by fitting the Nernst equation to the potential-dependent normalized area of ν_4_ (6cHS) at 1359 cm^−1^.

### 4.5. Redox Potential Determination in Solution

The potentiometric titration of CboDyP was performed in solution inside an anaerobic chamber (Coy Laboratory Products, Grass Lake, MI, USA) in an atmosphere of 95% Argon and 5% H_2_. The titration was monitored by UV–VIS spectroscopy using a UV–VIS Shimadzu 1800 spectrophotometer (Shimadzu, Kyoto, Japan). The solution potential was measured using a combined platinum–Ag/AgCl (3 M) reference electrode (Hamilton, Reno, NV, USA). CboDyP (10 μM) was in a de-aerated buffer (50 mM KPi and 75 mM NaCl, pH 7) containing each of the following mediators: duraquinone, menadione, indigo tetrasulphonate, indigo trisulphonate, phenazine, 2-hydroxy-1,4-naptoquinone, anthraquinone-2-sulphonate, safranine, benzyl viologen and methyl viologen. The concentration ratio between the enzyme and mediators was 2:1. The potential inside the cuvette was decreased by the stepwise addition of a buffered sodium dithionite solution, resulting in a change of the solution potential. Enzyme reduction was monitored by the evolution of the ferrous Soret absorption band at 430 nm, and the redox parameters were obtained by fitting the Nernst equation to the potential-dependent normalized absorption at 430 nm. The reversibility of the potentiometric titration was monitored by the stepwise oxidation of fully sodium dithionite-reduced CboDyP in the presence of mediators (*vide supra*) using potassium ferricyanide as the oxidant.

### 4.6. Electrochemistry Assays

CV and chronoamperometry experiments were performed in a SERR spectroelectrochemical cell. Prior to measurements, the supporting electrolyte (either 40 mM Britton–Robinson buffer and 50 mM KCl, pH 5 or 12.5 mM KPi and 12.5 mM K_2_SO_4_, pH 7) was deoxygenated by bubbling argon for 15 min; the cell was also maintained under argon atmosphere during the experiments. Cyclic voltammograms were recorded in the range of +300 to −200 mV at a scan rate of 50 mV s^−^^1^. Chronoamperometry experiments were performed at an applied potential of −100 mV with the Ag electrode rotating at 1500 rpm. To evaluate the response of the enzyme electrodes to H_2_O_2_, previously deoxygenated stock solutions (10 and 100 mM) were successively injected in the cell (final concentrations of H_2_O_2_: 8 μM to 1.7 mM). The catalytic currents (I_cat_) were corrected by subtracting the current measured in the absence of substrate. The concentrations of H_2_O_2_ in stock solutions were determined spectrophotometrically using a molar absorption coefficient of 43.6 M^−1^ cm^−^^1^ at 240 nm.

## Data Availability

Not applicable.

## Supplementary Materials

The following are available online at https://www.mdpi.com/article/10.3390/ijms22157998/s1, Figure S1: SERR spectra of ferric ScoDyP, TfuDyP and CboDyP, Figure S2: Electrochemical response of CboDyP.

## References

[1] Sugano Y. (2009). DyP-Type Peroxidases Comprise a Novel Heme Peroxidase Family. Cell. Mol. Life Sci..

[2] Colpa D.I., Fraaije M.W., Van Bloois E. (2014). DyP-Type Peroxidases: A Promising and Versatile Class of Enzymes. J. Ind. Microbiol. Biotechnol..

[3] Zhang R., Chen W. (2017). Recent Advances in Graphene-Based Nanomaterials for Fabricating Electrochemical Hydrogen Peroxide Sensors. Biosens. Bioelectron..

[4] Lippert A.R., Van de Bittner G.C., Chang C.J. (2011). Boronate Oxidation as a Bioorthogonal Reaction Approach for Studying the Chemistry of Hydrogen Peroxide in Living Systems. Acc. Chem. Res..

[5] Das P., Das M., Chinnadayyala S.R., Singha I.M., Goswami P. (2016). Recent Advances on Developing 3rd Generation Enzyme Electrode for Biosensor Applications. Biosens. Bioelectron..

[6] Bollella P., Medici L., Tessema M., Poloznikov A.A., Hushpulian D.M., Tishkov V.I., Andreu R., Leech D., Megersa N., Marcaccio M. (2018). Highly Sensitive, Stable and Selective Hydrogen Peroxide Amperometric Biosensors Based on Peroxidases from Different Sources Wired by Os-Polymer: A Comparative Study. Solid State Ion..

[7] Zuccarello L., Barbosa C., Todorovic S., Silveira C.M. (2021). Electrocatalysis by Heme Enzymes—Applications in Biosensing. Catalysts.

[8] Murgida D.H., Hildebrandt P. (2004). Electron-Transfer Processes of Cytochrome *c* at Interfaces. New Insights by Surface-Enhanced Resonance Raman Spectroscopy. Acc. Chem. Res..

[9] Murgida D.H., Hildebrandt P. (2001). Heterogeneous Electron Transfer of Cytochrome *c* on Coated Silver Electrodes. Electric Field Effects on Structure and Redox Potential. J. Phys. Chem. B.

[10] Sezer M., Millo D., Weidinger I.M., Zebger I., Hildebrandt P. (2012). Analyzing the Catalytic Processes of Immobilized Redox Enzymes by Vibrational Spectroscopies. IUBMB Life.

[11] Siebert F., Hildebrandt P. (2008). Vibrational Spectroscopy in Life Science.

[12] Khoa Ly H., Sezer M., Wisitruangsakul N., Feng J.-J., Kranich A., Millo D., Weidinger I.M., Zebger I., Murgida D.H., Hildebrandt P. (2011). Surface-Enhanced Vibrational Spectroscopy for Probing Transient Interactions of Proteins with Biomimetic Interfaces: Electric Field Effects on Structure, Dynamics and Function of Cytochrome c. FEBS J..

[13] Todorovic S., Jung C., Hildebrandt P., Murgida D.H. (2006). Conformational Transitions and Redox Potential Shifts of Cytochrome P450 Induced by Immobilization. J. Biol. Inorg. Chem..

[14] Todorovic S., Verissimo A., Wisitruangsakul N., Zebger I., Hildebrandt P., Pereira M.M., Teixeira M., Murgida D.H. (2008). SERR-Spectroelectrochemical Study of a *Cbb_3_* Oxygen Reductase in a Biomimetic Construct. J. Phys. Chem. B.

[15] Sezer M., Genebra T., Mendes S., Martins L.O., Todorovic S. (2012). A DyP-Type Peroxidase at a Bio-Compatible Interface: Structural and Mechanistic Insights. Soft Matter.

[16] Sezer M., Santos A., Kielb P., Pinto T., Martins L.O., Todorovic S. (2013). Distinct Structural and Redox Properties of the Heme Active Site in Bacterial Dye Decolorizing Peroxidase-Type Peroxidases from Two Subfamilies: Resonance Raman and Electrochemical Study. Biochemistry.

[17] Silveira C.M., Quintas P.O., Moura I., Moura J.J.G., Hildebrandt P., Almeida M.G., Todorovic S. (2015). SERR Spectroelectrochemical Study of Cytochrome Cd1 Nitrite Reductase Coimmobilized with Physiological Redox Partner Cytochrome C552 on Biocompatible Metal Electrodes. PLoS ONE.

[18] Silveira C.M., Castro M.A., Dantas J.M., Salgueiro C., Murgida D.H., Todorovic S. (2017). Structure, Electrocatalysis and Dynamics of Immobilized Cytochrome PccH and Its Microperoxidase. Phys. Chem. Chem. Phys..

[19] Todorovic S., Pereira M.M., Bandeiras T.M., Teixeira M., Hildebrandt P., Murgida D.H. (2005). Midpoint Potentials of Hemes *a* and *a_3_* in the Quinol Oxidase from *Acidianus ambivalens* Are Inverted. J. Am. Chem. Soc..

[20] Todorovic S., Rodrigues M.L., Matos D., Pereira I.A.C. (2012). Redox Properties of Lysine- and Methionine-Coordinated Hemes Ensure Downhill Electron Transfer in NrfH2A4 Nitrite Reductase. J. Phys. Chem. B.

[21] Barbosa C., Silveira C.M., Silva D., Brissos V., Hildebrandt P., Martins L.O., Todorovic S. (2020). Immobilized Dye-Decolorizing Peroxidase (DyP) and Directed Evolution Variants for Hydrogen Peroxide Biosensing. Biosens. Bioelectron..

[22] Wang Y., Wang Z., Rui Y., Li M. (2015). Horseradish Peroxidase Immobilization on Carbon Nanodots/CoFe Layered Double Hydroxides: Direct Electrochemistry and Hydrogen Peroxide Sensing. Biosens. Bioelectron..

[23] Zhang D., Zhao H., Fan Z., Li M., Du P., Liu C., Li Y., Li H., Cao H. (2015). A Highly Sensitive and Selective Hydrogen Peroxide Biosensor Based on Gold Nanoparticles and Three-Dimensional Porous Carbonized Chicken Eggshell Membrane. PLoS ONE.

[24] Villalonga R., Díez P., Yáñez-Sedeño P., Pingarrón J.M. (2011). Wiring Horseradish Peroxidase on Gold Nanoparticles-Based Nanostructured Polymeric Network for the Construction of Mediatorless Hydrogen Peroxide Biosensor. Electrochim. Acta.

[25] Pettersen E.F., Goddard T.D., Huang C.C., Meng E.C., Couch G.S., Croll T.I., Morris J.H., Ferrin T.E. (2021). UCSF ChimeraX: Structure Visualization for Researchers, Educators, and Developers. Protein Sci..

[26] Silveira C.M., Moe E., Fraaije M., Martins L.O., Todorovic S. (2020). Resonance Raman View of the Active Site Architecture in Bacterial DyP-Type Peroxidases. RSC Adv..

[27] Van Bloois E., Torres Pazmiño D.E., Winter R.T., Fraaije M.W. (2010). A Robust and Extracellular Heme-Containing Peroxidase from *Thermobifida fusca* as Prototype of a Bacterial Peroxidase Superfamily. Appl. Microbiol. Biotechnol..

[28] Habib M.H., Rozeboom H.J., Fraaije M.W. (2019). Characterization of a New DyP-Peroxidase from the alkaliphilic cellulomonad, *Cellulomonas bogoriensis*. Molecules.

[29] Bollella P., Gorton L. (2018). Enzyme Based Amperometric Biosensors. Curr. Opin. Electrochem..

[30] Monteiro T., Almeida M.G. (2019). Electrochemical Enzyme Biosensors Revisited: Old Solutions for New Problems. Crit. Rev. Anal. Chem..

[31] Brissos V., Tavares D., Sousa A.C., Robalo M.P., Martins L.O. (2017). Engineering a Bacterial DyP-Type Peroxidase for Enhanced Oxidation of Lignin-Related Phenolics at Alkaline PH. ACS Catal..

[32] Santos A., Mendes S., Brissos V. (2014). New Dye-Decolorizing Peroxidases from *Bacillus subtilis* and *Pseudomonas putida* MET94: Towards Biotechnological Applications. Appl. Microbiol. Biotechnol..

[33] Mendes S., Catarino T., Silveira C., Todorovic S., Martins L.O. (2015). The Catalytic Mechanism of A-Type Dye-Decolourising Peroxidase BsDyP: Neither Aspartate nor Arginine Is Individually Essential for Peroxidase Activity. Catal. Sci. Technol..

[34] Mendes S., Brissos V., Gabriel A., Catarino T., Turner D.L., Todorovic S., Martins L.O. (2015). An Integrated View of Redox and Catalytic Properties of B-Type PpDyP from *Pseudomonas putida* MET94 and Its Distal Variants. Arch. Biochem. Biophys..

[35] Smulevich G., Feis A., Howes B.D., Ivancich A. (2010). Structure-Function Relationships among Heme Peroxidases: New Insights from Electronic Absorption, Resonance Raman and Multifrequency Electron Paramagnetic Resonance Spectroscopies. Handbook of Porphyrin Science.

[36] Smulevich G., Feis A., Howes B.D. (2005). Fifteen Years of Raman Spectroscopy of Engineered Heme Containing Peroxidases: What Have We Learned?. Acc. Chem. Res..

[37] Battistuzzi G., Bellei M., Bortolotti C.A., Sola M. (2010). Redox Properties of Heme Peroxidases. Arch. Biochem. Biophys..

[38] Brown M.E., Barros T., Chang M.C.Y. (2012). Identification and Characterization of a Multifunctional Dye Peroxidase from a Lignin-Reactive Bacterium. ACS Chem. Biol..

[39] Chen C., Shrestha R., Jia K., Gao P.F., Geisbrecht B.V., Bossmann S.H., Shi J., Li P. (2015). Characterization of Dye-Decolorizing Peroxidase (DyP) from *Thermomonospora curvata* Reveals Unique Catalytic Properties of A-Type DyPs. J. Biol. Chem..

[40] Pfanzagl V., Nys K., Bellei M., Michlits H., Mlynek G., Battistuzzi G., Djinovic-Carugo K., Van Doorslaer S., Furtmüller P.G., Hofbauer S. (2018). Roles of Distal Aspartate and Arginine of B-Class Dye-Decolorizing Peroxidase in Heterolytic Hydrogen Peroxide Cleavage. J. Biol. Chem..

[41] Pfanzagl V., Bellei M., Hofbauer S., Laurent C.V.F.P., Furtmüller P.G., Oostenbrink C., Battistuzzi G., Obinger C. (2019). Redox Thermodynamics of B-Class Dye-Decolorizing Peroxidases. J. Inorg. Biochem..

[42] Kassner R.J. (1973). Theoretical Model for the Effects of Local Nonpolar Heme Environments on the Redox Potentials in Cytochromes. J. Am. Chem. Soc..

[43] Döpner S., Hildebrandt P., Mauk A.G., Lenk H., Stempfle W. (1996). Analysis of Vibrational Spectra of Multicomponent Systems. Application to PH-Dependent Resonance Raman Spectra of Ferricytochrome C. Spectrochim. Acta Part. A Mol. Spectrosc..

