# Supplementary material for: SERR Spectroelectrochemistry as a Guide for Rational Design of DyP-Based Bioelectronics Devices
Source: Int J Mol Sci. 2021 Jul 27;22(15):7998. doi: 10.3390/ijms22157998 (PMC8348443; doi:10.3390/ijms22157998)
Supplement: Supplementary file 1 [file ijms-22-07998-s001.zip › ijms-1296564-supplementary.pdf]

SUPPLEMENTARY MATERIAL

SERR spectroelectrochemistry as a guide for rational design of DyP based bioelectronics devices

Lidia Zuccarello, Catarina Barbosa, Edilson Galdino, Nikola Lončar, Célia M. Silveira, Marco W. Fraaije, Smilja Todorovic

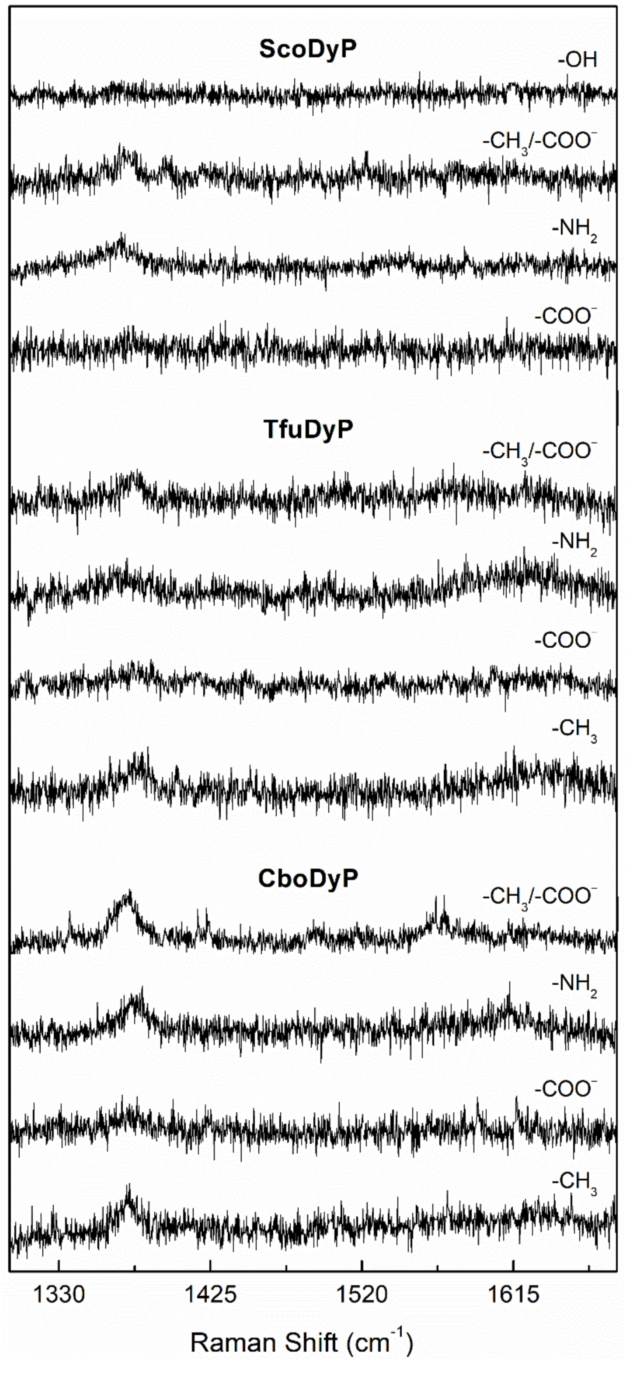

Figure S1. SERR spectra of ferric ScoDyP, TfuDyP and CboDyP immobilised on Ag electrodes coated with pure and mixed alkanethiol ($HS(CH_2)_n-X$, X = OH, CH_3, COO^- and NH_2) SAMs. All spectra were acquired with 405 nm excitation at 21 °C, pH 7.

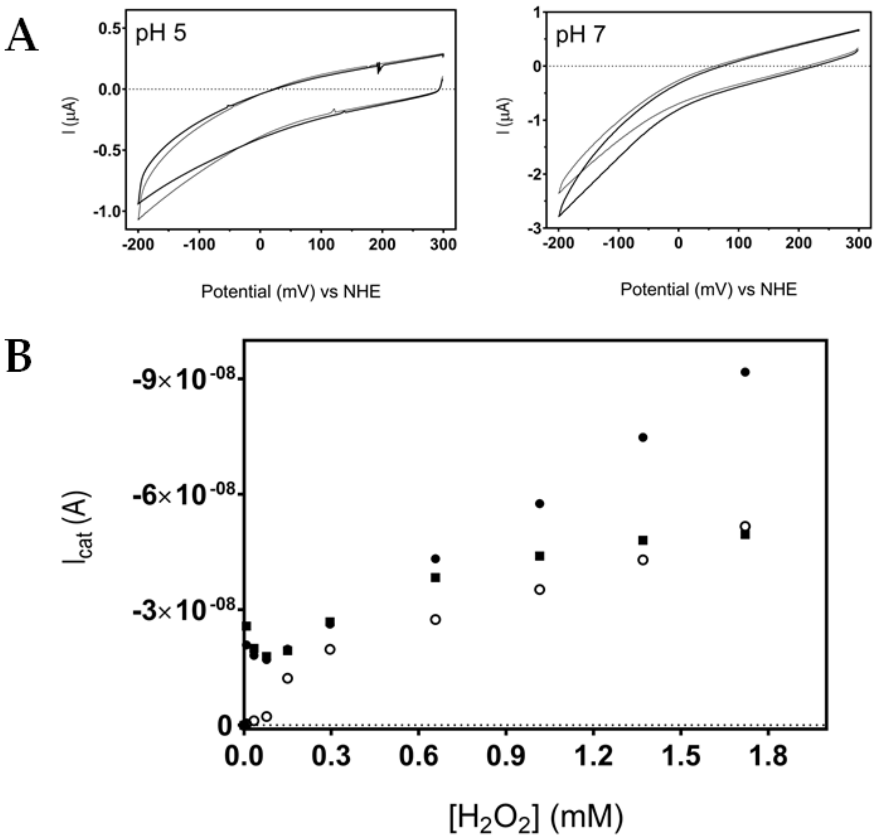

Figure S2. Electrochemical response of CboDyP immobilised on Ag electrodes coated with 1-undecanethiol : 11-amino-1-undecanethiol hydrochloride (M:M, 2:1) SAMs. (**A**) Cyclic voltammograms in absence (thin line) or presence (thick line) of H_2O_2, 0.7 mM, measured at pH 5 (Britton-Robinson 40 mM, KCl 50 mM) and pH 7 (KPi 12.5 mM, K_2SO_4 12.5 mM). (**B**) Plots of catalytic current (I_{cat}) vs. H_2O_2 concentration for CboDyP-loaded electrodes (full circles and full squares) and for control electrodes without enzyme (open circles); currents were measured by amperometry at −100 mV vs. NHE, pH 7.
